# Barriers to and Facilitators of Digital Health Among Culturally and Linguistically Diverse Populations: Qualitative Systematic Review

**DOI:** 10.2196/42719

**Published:** 2023-02-28

**Authors:** Lara Whitehead, Jason Talevski, Farhad Fatehi, Alison Beauchamp

**Affiliations:** 1 School of Rural Health, Faculty of Medicine, Nursing and Health Science Monash University Warragul Australia; 2 Institute for Physical Activity and Nutrition Research (IPAN), School of Exercise and Nutrition Sciences, Deakin University Geelong Australia; 3 Australian Institute for Musculoskeletal Science, The University of Melbourne and Western Health Melbourne Australia; 4 Centre for Health Services Research, Faculty of Medicine, The University of Queensland Brisbane Australia; 5 School of Psychological Sciences, Faculty of Medicine, Nursing and Health Sciences, Monash University Melbourne Australia

**Keywords:** culturally and linguistically diverse, ethnicity, indigenous, digital health, technology, eHealth, qualitative, mobile phone

## Abstract

**Background:**

Health care systems have become increasingly more reliant on patients’ ability to navigate the digital world. However, little research has been conducted on why some communities are less able or less likely to successfully engage with digital health technologies (DHTs), particularly among culturally and linguistically diverse (CaLD) populations.

**Objective:**

This systematic review aimed to determine the barriers to and facilitators of interacting with DHTs from the perspectives of CaLD population groups, including racial or ethnic minority groups, immigrants and refugees, and Indigenous or First Nations people.

**Methods:**

A systematic review and thematic synthesis of qualitative studies was conducted. Peer-reviewed literature published between January 2011 and June 2022 was searched across 3 electronic databases. Terms for digital health were combined with terms for cultural or linguistic diversity, ethnic minority groups, or Indigenous and First Nations people and terms related to barriers to accessing digital technologies. A qualitative thematic synthesis was conducted to identify descriptive and analytical themes of barriers to and facilitators of interacting with DHTs. Quality appraisal was performed using the Mixed Methods Appraisal Tool.

**Results:**

Of the 1418 studies identified in the electronic search, a total of 34 (2.4%) were included in this review. Half of the included studies (17/34, 50%) were conducted in the United States. There was considerable variation in terms of the CaLD backgrounds of the participants. In total, 26% (9/34) of the studies focused on Indigenous or First Nations communities, 41% (14/34) were conducted among ethnic minority populations, 15% (5/34) of the studies were conducted among immigrants, and 18% (6/34) were conducted in refugee communities. Of the 34 studies, 21 (62%) described the development or evaluation of a digital health intervention, whereas 13 (38%) studies did not include an intervention but instead focused on elucidating participants’ views and behaviors in relation to digital health. From the 34 studies analyzed, 18 descriptive themes were identified, each describing barriers to and facilitators of interacting with DHTs, which were grouped into 7 overarching analytical themes: using technology, design components, language, culture, health and medical, trustworthiness, and interaction with others.

**Conclusions:**

This study identified several analytic and descriptive themes influencing access to and uptake of DHTs among CaLD populations, including Indigenous and First Nations groups. We found that cultural factors affected all identified themes to some degree and that cultural and linguistic perspectives should be considered in the design and delivery of DHTs, with this best served through the inclusion of the target communities at all stages of development. This may improve the potential of DHTs to be more acceptable, appropriate, and accessible to population groups currently at risk of not obtaining the full benefits of digital health.

## Introduction

### Background

Over the past decade, the health care system has increasingly relied on digital technologies to educate, organize, and support people in managing their health [1]. Digital health can be described as “the field of knowledge and practice associated with the development and use of digital technologies to improve health” [2]. Patient-facing technologies such as telehealth systems, web-based services, or smartphone apps are now widely used to support the prevention, diagnosis, treatment, and self-management of health [3]. There is a large body of evidence demonstrating the effectiveness of digital health technologies (DHTs) for the treatment and prevention of various health conditions across multiple settings, including communities, primary care, and hospitals [4-6]. Furthermore, the use of DHTs has been shown to address other barriers that patients commonly experience with the health care system, such as poor access to health care providers and increased costs [7].

Despite substantial investment and an increasing number of DHTs available to health care consumers, their uptake has been limited among some population groups [8]. These include lower–socioeconomic-level groups and people from culturally and linguistically diverse (CaLD) communities, with the latter including racial or ethnic minority groups, immigrants and refugees, and First Nations people [8-15]. Studies have repeatedly reported that CaLD populations have lower uptake and use of DHTs than non-CaLD populations [8,16,17]. For example, US-based studies have found that African American and Latino people are less likely to use digital technologies for health care than White Americans [12,18], whereas other studies have shown less acceptability of DHTs among immigrants [12,13]. Indigenous and First Nations people are also less likely to use DHTs because of difficulties in access and low cultural appropriateness of the technology [19]. As such, DHTs may widen the existing inequalities within our health care system by creating a digital divide that affects the effective and equitable delivery of care [10].

This lower uptake of DHTs may, in part, be owing to a failure to meet the cultural, linguistic, or health literacy needs of these diverse population groups [20]. When designing DHTs, it is important to gain an understanding of the perspectives of the end users themselves (eg, consumers, patients, and carers) on the factors influencing their use of digital health [17]. Qualitative research approaches offer the opportunity to explore these perspectives in depth, with studies conducted among CaLD populations identifying several barriers to and facilitators of the uptake of digital health, including technical literacy, access to the internet, and acceptance of services [17]. Although this evidence is important for understanding barriers and facilitators from a consumer’s perspective, these qualitative studies are often small or focused on a single digital health intervention and, as such, may not provide representative data [21]. Furthermore, although existing qualitative reviews have explored barriers to and facilitators of the uptake of DHTs, few have explored the reasons influencing their use among CaLD populations [19,22-24].

### Objectives

This study aimed to review and synthesize qualitative literature to determine the barriers to and facilitators of interacting with DHTs from the perspectives of CaLD population groups. In this review, CaLD populations were defined as those who were (1) born in countries where the official language differs from that of their current country of residence or their language spoken at home is not the official language of the country where they reside, (2) First Nations or Indigenous populations (who may or may not speak English), or (3) populations who were described in studies as “ethnically/racially diverse” or “ethnic/racial minority” [11]. We acknowledge the considerable heterogeneity among these groups; however, our intention was to capture population groups that are generally underserved in the health sector based on their cultural, linguistic, or ethnic background to identify the appropriateness of digital health for these groups and highlight areas for improvement [19]. We also acknowledge a recent recommendation that First Nations or Indigenous populations be reported separately from CaLD findings in research projects [11]. However, the small sample size of many studies with First Nations or Indigenous people indicates a need for caution when drawing community-wide conclusions, as recommended in other studies [25,26]. Therefore, for the purposes of assessing this review, we grouped these populations as we hypothesized that they may share many of the same concerns in terms of barriers and facilitators regarding DHTs.

## Methods

### Study Design

A systematic review and thematic synthesis of qualitative studies were conducted following the guidance of the Enhanced Transparency of Reporting the Synthesis of Qualitative Research framework and reporting guidelines [27].

### Inclusion Criteria

Inclusion criteria were defined according to the Population, Intervention, Comparison, Outcome, and Study Type framework (Textbox 1). Additional inclusion criteria included publication in a peer-reviewed scientific journal in English and being accessible in full text. Protocols for research studies, book chapters, systematic reviews, and theses were excluded.

**Study inclusion criteria according to the Population, Intervention, Comparison, Outcome, and Study Type framework.**

- Population
  Studies on participants who were identified as patients, health consumers, or carers from culturally and linguistically diverse (CaLD) backgrounds or where CaLD groups were a subgroup of participants with results reported separately were included. To include studies from countries that do not share a common definition of CaLD communities, the following parameters were used to define CaLD: (1) those who were born in countries where the official language is not the same as that of their current country of residence or their main language spoken at home is not the official language of their current country of residence or (2) those described in the studies as “minority,” “ethnically/racially diverse,” and “ethnic/racial minority.” These studies may include Black and African American populations, as well as First Nations and Indigenous peoples in Canada, United States, New Zealand, and Australia.
- Intervention
  An intervention was included if it was delivered using a digital method (eg, smartphone) and directed at prevention, diagnosis, treatment, or self-management of health. Studies with no intervention were also included if participants discussed their perspectives on digital health more broadly as long as it was relevant to our aim. Studies conducted in any setting were included. Studies that focused only on telephone calls with no other digital health component were excluded.
- Outcomes
  Studies were included where findings were reported as participants’ perspectives on the barriers to and facilitators of access to and use of digital health or digital health technologies. Studies that reported clinician perspectives only were excluded.
- Study design
  Primary research studies were included if they used qualitative methods for data collection (eg, focus groups or interviews and open-ended survey questions) and data analysis (eg, thematic analysis). Studies using other designs (eg, mixed methods or cross-sectional) were included as long as there was a component of qualitative data collection and analysis that was reported separately from the quantitative component.

### Search Strategy

An electronic search using 3 web-based databases (Ovid MEDLINE, Embase, and CINAHL) was performed to find studies published between January 2011 and June 2022. Terms for digital health were combined with terms for cultural or linguistic diversity, ethnic minority groups, or Indigenous or First Nations people and terms related to barriers to accessing digital technologies. Ovid MEDLINE search terms are shown in Multimedia Appendix 1. Reference lists from eligible studies and systematic reviews were also searched. The results were imported into the Covidence systematic review software (Veritas Health Innovation), where each title and abstract was independently screened by 2 researchers, with discrepancies resolved by consulting a third researcher. Full texts of potentially relevant articles were then inspected for inclusion by 2 researchers based on the aforementioned inclusion criteria.

### Data Extraction

Data were extracted within Covidence using the following headings: study description (country of study and study design), participant description (percentage of female participants, mean age, and CaLD group), the type of digital health or DHT discussed and a brief description (if relevant), and results (extracted as relevant quotes from study participants and as text from the results section of each paper where authors identified barriers to and facilitators of interacting with DHT).

### Data Synthesis

We conducted a qualitative thematic synthesis as described by Thomas and Harden [28]. A thematic synthesis treats qualitative findings as data for analysis [28]; these data include both direct quotes from participants and summaries and interpretation of findings from study authors. We used an inductive approach, whereby themes related to interacting with digital health were identified from within the data. We followed the three thematic synthesis steps by Thomas and Harden [28]: (1) free line-by-line coding, (2) organization of these codes to construct descriptive themes, and (3) development of analytical themes. In step 1, quotes and text were coded (“named”) according to their meaning and content. One author (LW) coded all studies individually. New codes were added as necessary as the researcher continued re-examining the data. Two authors (LW and AB) discussed the codes and revised the coding schema as needed. In step 2, similar codes were grouped together to create descriptive themes related to barriers and enablers; at this point, the synthesis still followed the original findings of the included studies. The frequency of the themes was recorded as the number of times the theme was mentioned across the included articles. In step 3, these descriptive themes were interpreted to develop inferences about barriers and enablers and implications for future research (“analytical themes”). To examine the hypothesis that First Nations or Indigenous populations share many of the same barriers and facilitators regarding DHTs as other CaLD populations, we performed a subgroup analysis using the aforementioned data synthesis approach.

### Quality Appraisal

Quality appraisal was undertaken using the 2018 version of the Mixed Methods Appraisal Tool developed for use in systematic reviews of mixed methods studies [29]. This tool is designed for the appraisal stage of reviews that include qualitative, quantitative, and mixed methods studies and applies 5 specific quality criteria to each study design, with results presented as a percentage score for each study indicating the proportion of criteria met. For quantitative studies, quality criteria included randomization (if appropriate), representativeness of participants, completeness of outcome data, bias, confounding, and statistical analysis. For qualitative studies, quality criteria included appropriateness of the approach used; data collection and analysis methods; and coherence between data sources, analysis, and interpretation. For mixed methods studies, criteria were related to both the aforementioned quantitative and qualitative aspects plus the rationale for using a mixed methods design and adequate integration of the qualitative and quantitative phases of the study.

## Results

**Figure 1:**
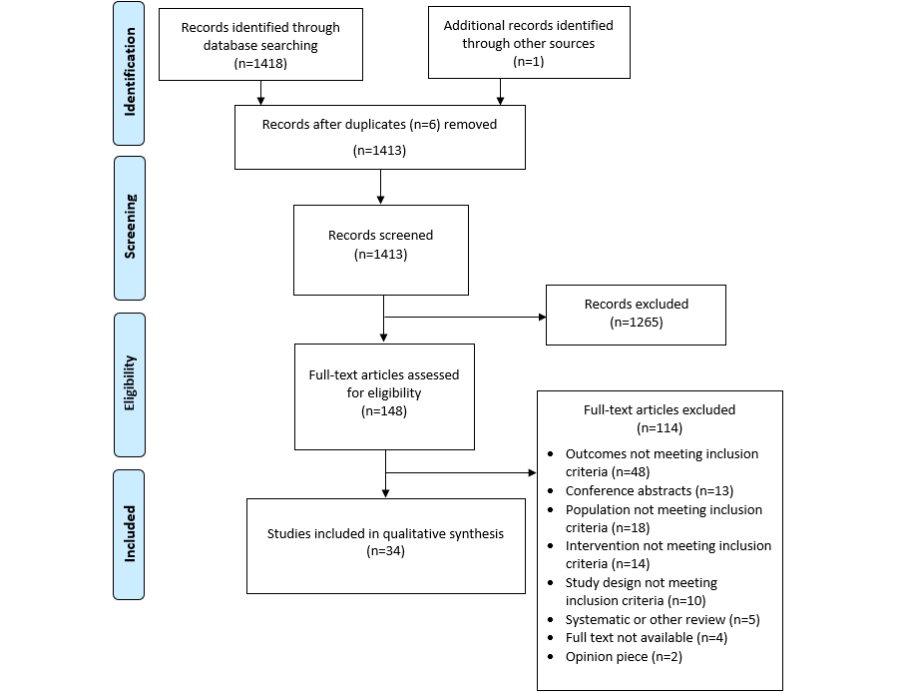
Prisma Flow Diagram.

A total of 1418 studies were identified through database searches, with 1 additional study identified through reference list searches. Of these 1419 studies, 148 (10.43%) full-text articles were assessed for eligibility, with 34 (2.4%) studies included in this review (Figure 1).

### Study Characteristics

Table 1 describes study and participant characteristics for all studies. Most of the included studies were conducted in the United States (17/34, 50%), Australia (6/34, 18%), and Canada (4/34, 12%), with 32% (11/34) of the studies using a mixed methods design and 6% (2/34) being randomized controlled trials, the latter including interviews with intervention participants to explore the cultural acceptability of the tool [30] and barriers to and enablers of the use of the app [31]. Nearly all studies (32/34, 94%) were conducted in the community, with 6% (2/34) conducted in primary care or outpatient clinics [32,33].

**Table 1:** Study and participant characteristics (N=34).

First author, year	Study design	Country and setting	Participant characteristics				Health condition		Quality rating
			CaLD^a^ background	Sample size, N	Age (years)				
Aguilera et al [34], 2014	Qualitative	United States; community	Latino, African American, and Euro-American	20	Mean 56	Depression		60%	
Bagchi et al [35], 2018	Mixed methods	United States; community	African American	10	Mean 59.9	None		60%	
Blackwell et al [36], 2020	Mixed methods	United States; community	African American, Hispanic, and Caribbean West Indian	56	FG^b^: range 20-45; T4B^c^: mean 28	Pregnancy		100%	
Brown et al [37], 2014	Qualitative	United States; community	African American and Hispanic	5	Mean 18.2 (SD 0.8)	Postpartum depression		80%	
Burchert et al [38], 2019	Qualitative	Egypt, Germany, and Sweden; community	Syrian refugees	128	I^d^: mean 33 (SD 11); KII^e^: mean 33.8 (SD 10.9); FG: NR^f^	Mental health		100%	
Claudel et al [39], 2020	Mixed methods	United States; community	African American	16	Mean 62.1 (SD 6.6)	Overweight or obesity		80%	
Filippi et al [40], 2012	Qualitative	United States; community	American Indians	108	NR	Smoking cessation		100%	
Garner et al [41], 2021	Mixed methods	Hong Kong; community	Asian Indian immigrants	46	Range 18-≥60; 54%, 15-59	Hypertension and diabetes		80%	
Gesser-Edelsburg et al [20], 2017	Qualitative	Israel; community	Former Soviet Union immigrants	18	Range 24-58	Nutrition and diet		80%	
Goodall et al [42], 2014	Qualitative	Australia; community	Greek and Italian immigrants	54	Mean 74.1	None		100%	
Hiratsuka et al [32], 2013	Qualitative	United States; primary care clinic	Alaska and Hawaiian Native	17	NR	Diabetes		80%	
Hyman et al [43], 2022	Qualitative	Canada; community	Asian Indian immigrants	46	Mean 65.4 (SD 12.1)	Chronic disease		100%	
Hynie et al [44], 2022	Qualitative	Canada; community	Syrian, Iranian, Eritrean, Somali, and Ethiopian refugees	197	Mean 35.4 (SD 9.8)	Mental health		80%	
Im et al [45], 2021	Qualitative	United States; community	Korean American	17	NR	Breast cancer		80%	
Johnson-Turbes et al [46], 2015	Mixed methods	United States; community	African American	180	NR	Breast cancer		80%	
Jongbloed et al [47], 2020	Mixed methods	Canada; community	First Nations, Inuit, and Métis	131	Mean 33 (range 30-36)	Illicit drug use		100%	
Kurth et al [30], 2016	RCT^g^	United States; community	Hispanic or Latino	61	Mean 48 (SD 12)	HIV		20%	
Lindegaard et al [48], 2021	Qualitative	Sweden; community	Arabic-speaking immigrants and refugees	10	Mean 33.4 (SD 9.1)	Mental health		80%	
Lindegaard et al [49], 2022	Mixed methods	Sweden; community	Dari or Farsi refugees and immigrants	7	Mean 22.3 (SD 2.35)	Mental health		60%	
Lyles et al [50], 2016	Qualitative	United States; community	African American and Hispanic or Latino	87	Range 24-69	Chronic disease		100%	
Maertens et al [51], 2017	Qualitative	United States; community	Hispanic or Latino	47	NR	HPV^h^		60%	
McAra-Couper et al [52], 2020	Qualitative	New Zealand; community	Mäori and Pacific Islanders	24	Range 16-40	Pregnancy and reproductive health		100%	
Merculieff et al [53], 2020	Mixed methods	United States; community	Alaska Native	30	Mean 45 (SD 12)	Smoking cessation		40%	
Ospina-Pinillos et al [54], 2019	Qualitative	Australia; community	Colombian, Chilean, Argentinian, Spanish, and Venezuelan immigrants	17	Co-design: mean 24 (range 17-29); think-aloud: mean 26 (range 19-30)	Mental health		60%	
Pasipanodya et al [55], 2020	Qualitative	United States; community	African American and Hispanic	13	Mean 46 (range 38-53)	HIV		100%	
Peiris et al [31], 2019	RCT	Australia; community	Aboriginal and Torres Strait Islanders	49	Mean 42 (SD 14)	Smoking		60%	
Povey et al [56], 2016	Qualitative	Australia; community	Aboriginal and Torres Strait Islanders	9	Mean 33 (SD 17)	Mental health		100%	
Rempel et al [57], 2016	Qualitative	Canada; community	First Nations, Inuit, and Métis	8	Mean 23	None		80%	
Spanhel et al [58], 2019	Qualitative	Germany; community	Syrian, Iranian, Eritrean, Algerian, and Iraqi refugees	6	Mean 38 (SD 11.8)	Sleeping issues		80%	
Tappen et al [59], 2021	Mixed methods	United States; community	African American, Hispanic, and European American	49	Mean 73 (SD 10)	None		80%	
Tighe et al [60], 2020	Mixed methods	Australia; community	Aboriginal and Torres Strait Islanders	13	Mean 24.2 (SD 4.7)	Mental health		60%	
Wan et al [61], 2018	Qualitative	United States; community	Tongan American	36	Mean 50	Physical activity		60%	
Willey et al [33], 2020	Qualitative	Australia; primary care	Afghan, Burmese, Indian, and Vietnamese refugees	22	NR	Mental health		100%	
Ye et al [62], 2012	Mixed methods	United States; community	Korean immigrants	16	Mean 45.1 (SD 8.2)	Mental health		20%	

^a^CaLD: culturally and linguistically diverse.

^b^FG: focus group.

^c^T4B: Text4Baby.

^d^I: interview.

^e^KII: key informant interview.

^f^NR: not reported.

^g^RCT: randomized controlled trial.

^h^HPV: human papillomavirus.

The sample sizes ranged from 5 to 197 (median 23), with an age range of 16 to 74 years. Most of the health topics considered were related to mental health (11/34, 32%), chronic health conditions (8/34, 24%), or lifestyle factors such as smoking or obesity and nutrition. In total, 12% (4/34) of the studies did not relate to any health topic. There was considerable variation in terms of the CaLD backgrounds of the participants. A total of 26% (9/34) of the studies focused on Indigenous or First Nations communities from the United States [32,40,53], Australia [31,56,60], Canada [47,57], and New Zealand [52]. Of the remaining 14 US studies, 3 (21%) were conducted solely in African American communities [35,39,46]; 2 (14%) were conducted among Hispanic or Latino groups only [30,51]; and 6 (43%) were conducted in mixed communities such as Latino, African American, and Euro-American communities [34,36,37,50,55,59]. Of the 17 US studies, 1 (6%) was conducted among Tongan American communities [61], and 2 (12%) were conducted among Korean immigrants or Korean American individuals [45,62]. Immigrants were also represented in 15% (5/34) of the studies from other countries, including Israel [20], Australia [42,54], Canada [43], and Hong Kong [41]. Refugees were represented in 6 studies: 2 (33%) from Sweden [48,49]; 1 (17%) each from Australia [33], Germany [58], and Canada [44]; and 1 (17%) conducted across Egypt, Germany, and Sweden [38].

### Study Quality

The quality rating for each study is shown in Table 1. A total of 65% (22/34) of the studies had a rating of ≥80%, indicating that they met at least 4 of the 5 criteria for that study design. Of the studies that met fewer criteria, reasons included limited description of the qualitative approach and methodology used by the qualitative designs. For quantitative designs, reasons included small or nonrepresentative samples and limited description of the tools used to assess outcomes.

### Description of Interventions

A total of 62% (21/34) of the included studies described a DHT (Table 2). Of these 21 interventions, 4 (19%) were classified as SMS text messaging services, including cognitive behavioral therapy for depression [34], health-promotion messages to new mothers [36,37], and checking participants’ use of illicit drugs [47]. In total, 29% (6/21) of the studies were classified as internet-based interventions; these included 67% (4/6) of mental health interventions [38,49,54,58], a series of skill-building videos for participants with HIV [30], and a multimedia intervention to improve sleep [58]. A total of 24% (5/21) of the interventions were classified as Android or iOS apps, including 20% (1/5) of the studies that evaluated 2 apps: a mental health intervention based on motivational intervention and a suicide prevention app [56]. A further study evaluated an app for improving mental health [60], and apps were also used to support smoking cessation [31] and teach hypertension and diabetes management [41]. One intervention was classified as a digital health survey offering screening and feedback for mental health issues before meeting with a midwife [33]. A total of 10% (2/21) of the interventions were classified as videoconference consultations; these included a telepsychiatry consultation [62] and a remote nursing assessment [35]. Other interventions included a web-based portal where patients could access their medical history and message their health care provider [50]. A total of 6% (2/34) of the studies used websites as their technology: one providing learning modules on breast cancer [46] and one tailored to users’ beliefs in relation to vaccination for human papillomavirus [51]. In total, 38% (13/34) of the studies did not include an intervention (data not shown). Instead, participants were asked for their views on mobile technology and health apps [39,61]; their web-based health information–seeking behavior and needs [40,42,52,57,59]; their use and intentions regarding telehealth [32]; their attitudes toward the use of apps in health-related research [55]; their views on health technology more broadly [43]; and their perspectives on internet-based mental health [44], social media postings [53], and technology-based cancer support programs [45].

**Table 2:** Description of each digital health intervention (N=21).

First author, year	Type of digital intervention and mode of delivery	Intervention description	Frequency
Aguilera et al [34], 2014	SMS text messaging service; mobile phone	SMS text messages as part of cognitive behavioral therapy for depression; patients received SMS text messages to aid in “homework,” consisting of monitoring of mood, thoughts, social interactions, and healthy activities	Four 4-week modules (16 weeks total)
Bagchi et al [35], 2018	Videoconference consultation; computer	A research nurse transported a laptop to a housing development to collect and transmit clinical information to a nurse practitioner off-site who could review clinical data and speak with patients and the nurse via live video stream	One 6-hour consult
Blackwell et al [36], 2020	SMS text messaging service; mobile phone	Text4baby—a mental health SMS text messaging service from the US Centers for Disease Control and Prevention that sends free SMS text messages to pregnant women or those with children aged <1 year providing information and reminders to improve health	4 weeks
Brown et al [37], 2014	SMS text messaging service; mobile phone	Health-promotion information in the form of text blasts or pictures sent to mothers during the first 6 months postpartum; topics included promotion of breastfeeding, information about infant immunizations, and reminders about infant and maternal follow-up and well-being	6 months (weekly)
Burchert et al [38], 2019	Internet-based intervention; computer or smartphone	SbS^a^—an e–mental health intervention for depression with 3 tailored components: the content (educative narratives and exercises), the guidance model (contact with a trained nonspecialist), and the delivery system (web or app)	NR^b^
Garner et al [41], 2021	Android or iOS app; smartphone or tablet	3D animation to teach hypertension and diabetes prevention and management; culturally tailored animated videos, short pre- and posttests, and brief educational games that included voice-over and icons	Once
Gesser-Edelsburg et al [20], 2017	Web-based forum; computer	Interviews to explore attitudes about the internet as a tool for nutritional therapy interventions; all participants were members of a web-based nutrition forum receiving nutritional consultation and long-term treatment via the internet	NR
Johnson-Turbes et al [46], 2015	Self-navigated website; computer	Self-navigated web-only interface with information about breast cancer grouped into an 8-section workbook presented on the site and downloadable in full or by section	NR
Jongbloed et al [47], 2020	SMS text messaging service; mobile phone	Weekly SMS text messages “checking-in” for young Indigenous people who have used drugs, with follow-up support from a case manager for participants reporting a problem	NR
Kurth et al [30], 2016	Internet-based intervention; computer	A computer-based counseling tool that includes audio narrated risk assessment, tailored feedback through skill-building videos, and a risk-reduction plan; on completion, participants received a printout of their tailored feedback and health-promotion plan to share with their health care provider	45-60 minutes
Lindegaard et al [48], 2021	Internet-based intervention; computer or smartphone	Individually tailored, guided ICBT^c^ treatment that was culturally adapted for the target audience: a total of 9 treatment modules and weekly feedback on homework, modules tailored based on participants’ responses, and email contact with therapists	8 weeks
Lindegaard et al [49], 2022	Internet-based intervention; computer or smartphone	An adapted version of a previously developed ICBT intervention for adult Arabic-speaking immigrants and refugees (see Lindegaard et al [48]); this adaptation included translating all treatment materials into Dari and Farsi, simplifying the language used to make it more suitable for a young population, and adding an extra module regarding prolonged grief and separation anxiety	8 weeks
Lyles et al [50], 2016	Web-based portal; computer or smartphone	The portal allows patients access to several features: (1) viewing medical history, including visit summaries and immunizations; (2) viewing laboratory results; (3) refilling medications; (4) making appointments; and (5) sending a secure message to a health care provider	NR
Maertens et al [51], 2017	Customized website; computer	Participants complete a survey, after which they are directed to customized web pages, including photographs to match demographics; prioritizing vaccine information that matches participants’ beliefs; referencing characteristics such as first name, age, and gender throughout the web pages; and using statements to reiterate participants’ stated opinions	NR
Ospina-Pinillos et al [54], 2019	Internet-based intervention; computer	MHeC^d^ for young people experiencing mental health problems; a total of 5 main elements: a home page with a triage system, a web-based physical and mental health self-report assessment, a results dashboard, a videoconferencing system, and a personalized well-being plan	NR
Peiris et al [31], 2019	Android or iOS app; smartphone or tablet	Multifaceted smoking cessation app comprising a personalized profile and quit plan, motivational in-app and SMS text messages, and a challenge feature allowing users to compete with others	3 times per user (average)
Povey et al [56], 2016	Android or iOS app; smartphone or tablet	Therapist-supported mental health intervention integrating motivational interviewing and cognitive behavioral therapy techniques; the app uses colorful graphics, audio, and animation with limited text; care plans can be saved, emailed, printed, and reaccessed as an ongoing monitoring tool	NR
Povey et al [56], 2016	Android or iOS app; smartphone or tablet	The i-bobbly suicide prevention app includes 3 modules; self-assessment modules ask the user if they are experiencing intrusive thoughts or thoughts of suicide; if so, they are directed to seek urgent help; activity modules use activities, stories, and videos to help users manage upsetting thoughts and emotions and set small, realistic goals	NR
Spanhel et al [58], 2019	Internet-based intervention; computer or smartphone	The eSano Sleep-e intervention—3 web-based modules using text and multimedia components (images, audios, and videos) as well as reports from role models and elements such as quizzes, a sleeping diary, and homework	3 modules (45 minutes each)
Tighe et al [60], 2020	Android or iOS app; smartphone or tablet	Used the i-bobbly suicide prevention app (see Povey et al [56])	3 modules over 6 weeks
Willey et al [33], 2020	Web-based screening survey; tablet	At the first antenatal visit, women were provided with an iPad and asked to complete the Edinburgh Postnatal Depression Scale and a psychosocial assessment; a report was sent to the women via email with their scores and links to further information; scores and reports were also available to the midwife for the consultation	One-time survey
Ye et al [62], 2012	Videoconference consultation; computer	A telepsychiatrist-conducted assessment and consultation via videoconference during which a facilitator remained in the waiting room in case patients needed assistance; when the telepsychiatry session ended, the facilitator re-entered the room and received the treatment plan and instructions on medication prescribed, future appointments, and any recommended referrals or resources	Weekly (over 20 weeks)

^a^SbS: step-by-step.

^b^NR: not reported.

^c^ICBT: internet-based cognitive behavioral therapy.

^d^MHeC: mental health e-clinic.

### Thematic Analysis Results

#### Overview

From the 34 studies analyzed, 20 descriptive themes related to barriers and facilitators were identified. From these, 7 overarching analytical themes were developed (Table 3). These were (1) using technology, (2) design components, (3) language, (4) culture, (5) health and medical, (6) trustworthiness, and (7) interaction with others. The subgroup analysis of studies conducted with First Nations or Indigenous populations identified the same descriptive and analytical themes, although the proportion of studies within each theme varied. As such, findings for all CaLD groups were combined for reporting purposes, with any differences observed for First Nations or Indigenous populations highlighted in the text.

**Table 3:** Analytic and descriptive themes.

Analytic and descriptive theme		Barriers (number of studies)	Facilitators (number of studies)
**Using technology**			
	Digital literacy	Limited skills to use technology (16) Searching for credible information (4) Learning new skills (1) Fear of technology (3)	Education for digital literacy (5) Familiarity with phones (3) Help from family (1)
	Accessibility	Cost of phones and data (10) Poor functionality of digital services (10) Limited user-friendly technology (6) Gender-related factors (2)	Less travel and costs (11) User-friendly technology (3) Accessible via mobile phones (3) Availability of public internet (2) Improves access to care for disadvantaged groups (2)
	Interaction with digital systems	—^a^	Interaction easier via computer (2) Computers are nonjudgmental (2)
**Design components**			
	Content	Repetitive (5) Not engaging (2) Overall length (4)	Games and interactivity make content engaging (5) Use of graphics (3) Ensure relevant storyline (2)
	Delivery of information	Limited user control (3)	Convenient and time-saving (7) Quick access to information and health care providers (5) Timely reminders (3)
	End-user input	Lack of consultation with end users (1)	Value of co-design or user engagement (1)
**Language**			
	Literacy in the English language	Limited English literacy (11) Complex terminology (4)	Use strategies to support users with low English literacy (5)
	Literacy in first language	Limited literacy in first language (4)	Audio in first language for low literacy (1)
	Preference for own language	Lack of native language (4)	Use of native language (10)
**Culture**			
	Cultural representation	Lack of cultural representation (4)	Cultural diversity is well represented (3)
	Cultural appropriateness	Limited recognition of cultural concerns (5) Cultural norms not considered (2) Limited appropriateness for refugees (2)	Content and approach relevant to culture (7) Cultural norms considered (2)
**Health and medical**			
	Standard of care	Lower standard of care (2) No physical assessment (2)	Personalized care (3) Modern approach (1)
	Health information	Nonspecific focus (3) Supplementary only (1)	Positive health impacts (2)
	Psychosocial aspects	Emotions and mood affect uptake (2) Adverse psychological outcomes (4)	Empowerment and independence (5) Positive psychological outcomes (4)
**Trustworthiness**			
	Privacy and confidentiality	Privacy from others (5) Safety of personal information (5)	Greater privacy is valued (9)
	Information reliability	Distrust of web-based information (5)	Trust in information (1) Confidence in critical appraisal skills (1)
**Interaction with others**			
	Interaction with health care providers	Lack of personal connection affects relationship (9)	—
	Social connectedness	—	Builds social connections (7) Increases social support (3) Learning from others (1)

^a^No barriers and facilitators for that particular theme.

#### Using Technology

In total, 3 descriptive themes influenced the way in which people used technology for health.

##### Digital Literacy

Digital literacy is defined as the skills necessary for technology use and problem-solving [63]. Core digital skills such as the ability to open an app, use SMS text messaging, or manually enter data proved challenging for many participants and, in some cases, created a reliance on others for help [39,43]. This finding was observed across all cultural groups and ages and in 38% (13/34) of the studies [30,31,34,35,38,39,42,43,50,​55-57,59,61,62,64]. Other digital literacy barriers included a fear that technology is dangerous or intimidating reported by older Greek and Italian immigrants [42], Indigenous people [47], and Asian Indian immigrants [43]. Challenges with searching for credible and understandable web-based information were noted, including a self-reported lack of skills and confidence and feeling overwhelmed by the complexity of the information available [20,43,52,57].

Conversely, 9% (3/34) of the studies reported that familiarity with the use of mobile phones facilitated digital literacy [36,37,62]. Education was also mentioned as a facilitator for addressing low digital literacy, including attending classes held by ethno-specific service providers. This suggestion was reported among older African American, Hispanic, and European American individuals in the United States [39,59]; older Greek and Italian migrants in Australia [42]; younger Indigenous people in Canada [57]; and older Asian Indian immigrants in Canada [43], although the effort and cost associated with learning new skills were highlighted among the latter group.

##### Accessibility

Poor functionality of digital services was described as a barrier in 29% (10/34) of the studies across a range of cultural groups [30-32,35,38,39,48,49,59,62]. Specific barriers included issues with connectivity and reliability of the internet, often resulting in frustration on the part of the user. No facilitators were identified for functionality.

The cost of purchasing phones and data was also reported as a barrier to access across a range of cultural groups and ages [38,42,44,47,52,55,56,59], although 6% (2/34) of the studies identified that this could be partly outweighed by using public internet services such as those available in community libraries [40,52]. For American Indian individuals, lack of internet on reservations was also highlighted [40], and for Aboriginal Australians, DHTs were thought to be not always appropriate for people living in remote communities [56]. The cost savings associated with DHTs were seen as facilitators across most cultural groups, including fewer health care costs and less need for travel [20,32,35,38,39,44,45,47,60,62,64]. Other accessibility-related barriers reported across various cultural groups included limited user-friendly technology for older people [38,42,56] and the use of small screens or text [34,39,47]. Gender-related factors were also noted, such as domestic priorities for Asian Indian women [43] and inequities in access to technology for women refugees [44]. A total of 9% (3/34) of the studies on participants across a wide age range identified that mobile apps were more accessible than websites because of convenience and the fact that they use their phones “all day, every day” [39,50,54]. In total, 6% (2/34) of the studies noted that digital technologies could improve access to limited resources (eg, first-language therapists) [44], including for rural populations [49].

##### Interaction With Digital Systems

Some participants identified that talking with a computer was easier than talking to a person face to face, reporting that apps or computers are not judgmental. These studies were conducted with Aboriginal and Torres Strait Islanders [56], Mäori and Pacific Islanders [52], refugees [33], and South American migrant communities [54]. All these studies discussed user perspectives on mental health interventions. There were no reported barriers for this descriptive theme.

#### Design Components

##### Content

The importance of having engaging content was highlighted in 32% (11/34) of the studies. DHTs that were repetitive or had low visual appeal or limited interactivity were seen as frustrating or boring in 21% (7/34) of the studies that spanned all ages and cultural groups [31,33,38,41,46,59,60]. Across a range of cultural groups, participants suggested that apps could be made more engaging by incorporating games, challenges, and social elements [31,41,46,53,61], with the latter thought to also increase social connectedness among Aboriginal and Torres Strait Islanders [31]. In total, 9% (3/34) of the studies reported that the use of plain language, bullet points, and graphics would help users stay engaged with a website [40,41,53]. Incorporating a purposeful and culturally relevant storyline where individuals were virtually supported through a journey was also highlighted as important to Indigenous people in Australia [56] and the United States [53].

Having a large amount of content to read was also seen as a barrier to engagement with DHTs by younger refugees [38,58], African American individuals [46], and Asian Indian immigrants [41].

##### Delivery of Information

Studies among African American, Hispanic, and Latino participants identified that not being able to control the delivery of information could be frustrating, including poor timing of SMS text messages [34,55] and having little control over the amount of information provided at any one time [51]. Conversely, some participants reported the timeliness of messages as a facilitator, either as a reminder for managing health or as a motivating factor [31,34,46]. Delivery of health care or information via digital means was also thought to enable quick access, including immediate access to information and care and reducing the need to make appointments. This finding was observed across a range of cultural groups [20,35,52,56,60,64]. Convenience was also a facilitator of the uptake of DHTs, with many participants reporting less time spent in clinics and flexibility of access at a time and location to suit the end user [35-38,43,47,48].

##### End-User Input

Lack of end-user contribution to the design of a smoking cessation app was seen as a barrier to engagement in 3% (1/34) of the studies [31], whereas community involvement in app development was highlighted as a potential facilitator [56]. Both of these studies were conducted within Aboriginal and Torres Strait Islander communities.

#### Language

##### Literacy in the English Language

A common problem reported across many cultural groups, including refugees, was that of limited English literacy [33,36,42,46,52,54,56,57,59]. This was made more challenging by an overwhelming amount of text [38,51] and the use of complex medical terminology in some applications [30,39,48,52], including government health websites, as reported by Native American individuals [40]. Recommendations to support users with low literacy included the increased use of pictures, videos, and explanations and greater use of plain language, as reported by refugees [38,58], Indigenous people [40,56], and Asian Indian immigrants [41].

##### Literacy in the First Language

A total of 9% (3/34) of the studies reported that illiteracy in their own language was an issue for refugee groups [33,38,58] and Asian Indian immigrants [41]. The use of audio versions was suggested by refugees as a way of supporting access to information for people with limited ability to read in their native language [33].

##### Preference for Own Language

Numerous studies (10/34, 29%) across a wide range of cultural groups reported a strong preference for digital applications to be accessible in the first language of the participants [20,30,33,38,43,45,48,54,56,62] rather than in English or other dominant languages. A study on refugee Syrian women noted their praise for the use of their vernacular form of Levantine Arabic rather than formal Arabic in a mental health support app [38]. Having a clinician who spoke the patients’ own language during telehealth appointments was also thought to improve interaction with health care providers among Asian American individuals and Korean immigrants [62] and immigrants from the former Soviet Union [20]. Overall, perceptions were that this would support greater usability, including for Aboriginal and Torres Strait Islanders, who noted that this would increase their engagement with a mental health intervention [56].

#### Culture

##### Cultural Representation

Of the 34 studies, 2 (6%) conducted among refugees [38,58] and 2 (6%) conducted among Indigenous people [40,53] reported that seeing familiar, “contemporary” examples of their culture represented visually, with sensitivity and accuracy, was extremely important and influenced their desire to engage with the technology. This included using narrators from the same culture and background [38,53].

##### Cultural Appropriateness

Lack of recognition of cultural concerns was reported as a barrier to the use of DHTs in 15% (5/34) of the studies [30,39,45,51,58]. Of these 5 studies, 2 (40%) were conducted among Hispanic and Latino participants, and barriers included not identifying the issues faced by the community [51] and the use of culturally inappropriate questions [30]. American Indian individuals emphasized the importance of avoiding stereotyping or inaccurate portrayals of modern native people as this was seen as disrespectful [40]. The need to be aware of underlying community issues was also raised by African American participants [39]. Facilitators for cultural appropriateness included ensuring that the content and approach are relevant to the cultural community, such as using stories that reflect their experience, or ensuring that any health care providers are of the same cultural background [20,38,39,53,56,61,62]. Among refugees, the importance of avoiding the triggering of past traumas through inappropriate images (such as boats) within a DHT was highlighted, as well as understanding the restrictions of their current circumstances in being able to engage fully with an intervention [58]. A study also reported concerns about the acceptability of certain topics for refugees [48]. Cultural norms regarding electronic versus face-to-face communication were also highlighted in studies on Asian Indian immigrants [43] and Indian refugees [44], with both cultural groups noted to have a strong oral tradition.

#### Health and Medical

##### Standard of Care

There was a perception among some participants in the United States and Canada that delivery of health care via digital means led to a lower standard of care, including no opportunity for a physical assessment [32,35,44]. Conversely, several studies (4/34, 12%) across a range of cultural groups reported that the use of digital technologies allowed for an improved standard of care that was more personalized and up-to-date [45,55,56,59]. Among Alaska and Hawaiian Native participants, telehealth was thought to be improved if supported by an initial face-to-face visit with the clinician to allow for a thorough clinical assessment [32].

##### Health Information

Perceptions of information-based resources such as those used to promote vaccinations included an overfocus on medical information, with some African American and Hispanic and Latino participants wanting a greater focus on health and wellness [51,55] and others wanting more personalized information [39]. Both Indian-Asian migrants [43] and Korean American individuals [45] reported that web-based health information could lead to improved health.

##### Psychosocial Aspects

Among refugee communities, psychosocial barriers to the uptake of DHTs included existing psychological distress [38] or fatigue [58]. The risk of adverse psychological outcomes from using a DHT was also a potential barrier across several cultural groups, including becoming distressed [55,60] and feeling demotivated after receiving negative feedback from the DHT, such as automated comments appearing after failing a quiz [31,58]. Several studies (3/34, 9%) identified positive psychosocial outcomes from the use of DHTs, such as greater personal and psychological support [20,38] and increased awareness of mental health issues [54].

#### Trustworthiness

##### Privacy and Confidentiality

Privacy was both a barrier to and enabler of the use of DHTs for many cultural groups. Privacy from others when using computers or mobile phones was reported as a barrier in 15% (5/34) of the studies [33,35,44,50,55], including the need to remain private to avoid judgment from others [55]. Of note, 60% (3/5) of these studies were conducted among African American participants [35,50,55], with one author identifying that privacy is an important issue for this population [50]. Loss of privacy when using interpreters to help translate digital health information was noted to potentially lead to loss of truthfulness and avoidance of culturally taboo subjects among refugees [33]. Other participants, primarily those from Indigenous, refugee, or African American communities, felt that DHTs improved their sense of privacy and valued the anonymity they provided [30,35,47-49,52,55,56,60]. For example, Aboriginal participants in Australia expressed the idea of culturally based “shame” when addressing mental health issues inside their communities and noted the privacy that the digital app afforded [60]. Safety of personal information was also a frequently cited barrier across many cultural groups, with concerns about the trustworthiness of the technology, the risk of websites being “hacked,” and not being able to verify who is at the other end [38,39,47,52,55].

##### Information Reliability

Several studies (3/34, 9%) reported a lack of trust in web-based information, including in its currency and reliability [38,40,43,57]. In terms of believability, Aboriginal participants felt that a suicide prevention app’s claim of its impact was overstated [56]. A study among Hispanic and Latino participants reported that a digital health intervention to promote the uptake of the human papillomavirus vaccine provided reliable information and was similar to having access to another physician [30]. Among Korean American participants, confidence in critical appraisal skills was reported as important for judging information reliability [45].

#### Interaction With Others

##### Interaction With Health Care Providers

Across a number of cultural groups, DHTs were considered to adversely affect participant-provider interaction as they were described as being impersonal in nature, affecting communication, motivation, and understanding [35,38,44,48-50]. The DHTs discussed included videoconference consultation, mental health interventions, and a patient portal. The limited personal connection inherent in most DHTs was considered to negatively affect the ability to build good relationships with providers in 9% (3/34) of the studies [32,50,62].

##### Social Connectedness

This descriptive theme included 3 facilitators of the uptake of DHTs observed across a range of cultural groups. These were an increased sense of social support through web-based social interactions such as chat rooms [36,37,45]; the ability to learn from others who were experiencing the same issue [36]; and the ability to build social connections with other users [30,34,37,47,55,59,61], including through being able to reach out to others more easily, noted among First Nations, Inuit, and Métis participants [47].

## Discussion

### Principal Findings and Comparison With Other Studies

This study identified several analytic and descriptive themes influencing access to and uptake of DHTs among CaLD populations, including Indigenous groups. We found that cultural factors affected all the identified themes to some degree and that cultural and linguistic perspectives should be considered in the design and delivery of all DHTs, with this best served through the inclusion of the target communities at all stages of development.

The dominant analytic theme identified in this review was “Using Technology,” with accessibility and digital literacy (the skills required for technology use) being the most common barriers observed across most cultural groups. Although both factors are also noted as barriers to the uptake of DHTs among the wider population [21], this review suggests several mitigation strategies for culturally diverse populations. These include the delivery of DHTs via socially normative and familiar technologies (eg, mobile phones) and the provision of culturally tailored education programs. Improving access to free internet in public spaces (eg, community libraries) may also be a useful strategy. This access should address concerns about privacy noted among some population groups such as African American individuals [65] and also the irregular availability of public internet services [10,66]. The second most frequent analytic theme was “Language,” and for many participants across a range of cultural groups, lower literacy in English or in their own language was a barrier to the use of DHTs. This finding has also been observed in other systematic reviews [21]. The use of good health literacy principles when designing DHTs, such as plain language, limited text, clear layout, and use of appropriate images, would go some way toward addressing these language issues [24]. The delivery of content through multiple modes (audio, video, and text) could also enable users to understand basic messages without language or literacy barriers [22]. The use of vernacular languages within DHTs could also be considered as this may be more understandable to many users [67]. The theme of “Culture” identified the importance of ensuring that community sensitivities are considered and highlighted the value of seeing one’s own culture accurately represented. Although this analytic theme was less frequent than others, cultural-specific factors were noted within many other themes, such as fear of technology among Greek or Italian migrants and Indigenous people, triggering of past traumas through culturally inappropriate images for refugees, and the importance of co-design with Indigenous people. Finally, trustworthiness was also a common theme, including distrust of web-based information. There is limited literature on cultural or linguistic factors influencing trust in web-based information even in relation to the COVID-19 pandemic. Some comparative studies have suggested that cultural theories may underpin differences in trust between different populations (eg, individual vs collective societies) [68,69], whereas others report that an underlying mistrust of the medical system may also influence attitudes toward web-based health information [70,71]. This is an area requiring further research, including whether DHTs that are linguistically or culturally appropriate are seen as more trustworthy and also whether alternative strategies such as having community leaders champion the use of DHTs may be effective at enhancing cultural relevance [72].

### Implications for Future Research Directions

Overall, there are considerable gaps in the evidence base for factors influencing interaction with DHTs among CaLD populations, including from the perspectives of community members [8,23,73]. This is surprising given the substantial investment in digital health in recent years [8]. These gaps include lack of evidence on health literacy and digital health literacy in CaLD populations, both of which are prerequisites for the effective use of DHTs. CaLD populations have been shown to have relatively low health literacy [74,75], whereas digital health literacy is rarely reported [23,76], although some European studies suggest it is lower among migrants and non–native language speakers [77,78]. Inadequate digital health literacy is compounded by barriers related to internet access, cultural and linguistic factors, self-efficacy in using digital technology, and concerns about privacy, all of which were factors identified in this review. Disparities in digital health literacy may lead to further widening of existing health inequalities, and there is a need to ensure that digital technologies are accessible and usable by all [10,65]. Therefore, a greater understanding of the digital health literacy needs of CaLD groups is required [23].

There are also few studies demonstrating the input of CaLD community members themselves during the development of DHTs. Participatory-based approaches such as co-design may be warranted to ensure that DHTs meet the needs of CaLD groups [79]. Co-design is a process in which targeted end users, stakeholders, and researchers work together on all aspects of intervention development, from needs assessment to content development and pilot-testing. In a recent meta-analysis, co-designed interventions were shown to have positive effects on health behaviors across multiple health conditions [80]. However, co-design of health technologies with culturally diverse groups is an emerging area [39,54,81,82], and there is little evidence on involving CaLD communities in the co-design of DHTs. A systematic review of eHealth interventions targeted at socially disadvantaged groups (including ethnic minority groups) identified that most interventions did not involve members of these communities in their development [76].

A further area for future research is understanding the factors influencing the digital divide. People from CaLD and First Nations or Indigenous communities are more likely to experience socioeconomic disadvantage [11], which will only compound many of the challenges associated with the use of DHTs identified in this review and further exacerbate the digital divide [17]. However, it is noteworthy that 15% (5/34) of the included studies highlighted participants’ motivation to engage with DHTs, including a stated willingness to improve their digital literacy skills [43]. Studies also highlighted the widespread use of mobile phones. Therefore, it is important that efforts to reduce the digital divide for CaLD populations do not just focus on access to technologies or assume a lack of motivation to adopt them. Rather, a greater focus on appropriate tailoring through understanding the cultural needs and assets within communities and then co-designing solutions is warranted [17].

Finally, it would be valuable to explore potential interactions between themes to enhance the value and effectiveness of DHTs. As noted previously, culture was a factor that influenced many of the themes identified in this study; however, the findings also suggested other interactions, such as using design elements to improve both social connectedness and engagement with content and the importance of a common language in creating positive interactions with clinicians during telehealth appointments.

### Strengths and Limitations

This is the first qualitative systematic review to focus on the perspectives of CaLD populations. We used a comprehensive search strategy and a systematic, widely used method for synthesizing qualitative data. However, the limitations of this review must also be discussed. There was no consistent definition of CaLD populations in each of the reviewed articles, which might limit the generalizability and comparison of the findings [11]. The review included a broad range of cultural, linguistic, ethnic, and Indigenous groups that are heterogeneous with respect to multiple factors. Where possible, findings were reported separately to highlight barriers and facilitators unique to each population group and those that were common across several groups. Search terms for facilitators were not included. In addition, we were unable to contact the authors of the 12% (4/34) of the studies where full text was not available. As such, we may have missed some evidence in this review.

### Conclusions

The combination of a health system that relies heavily on a patient’s skills in navigating the largely English-based digital system, with substantial proportions of the population for whom English is not their native language, leaves a potential gap in the ability of policy makers and health care providers to deliver health care in an equitable and effective manner. As technological innovations continue to become inseparable from health care, a greater understanding of the needs of CaLD populations will identify how digital health can be tailored to support improved health outcomes in these disadvantaged groups. The inclusion of CaLD community members in the design and development of DHTs may help ensure that cultural, linguistic, and other factors are considered and addressed from the outset. In turn, this may improve the potential of DHTs to be more acceptable, appropriate, and accessible to population groups currently at risk of not obtaining the full benefits offered by digital health.

## Appendix

Multimedia Appendix 1 Search terms.

## Abbreviations

CaLD: culturally and linguistically diverse
DHT: digital health technology
