# Supplementary material for: Barriers to and Facilitators of Digital Health Among Culturally and Linguistically Diverse Populations: Qualitative Systematic Review
Source: J Med Internet Res. 2023 Feb 28;25:e42719. doi: 10.2196/42719 (PMC10015358; doi:10.2196/42719)
Supplement: Multimedia Appendix 1 [file jmir_v25i1e42719_app1.docx]

| **Search terms used in MEDLINE** |
| --- |
| Ovid MEDLINE(R) <1946 to June 29, 2021>  1 exp telemedicine/ or exp remote consultation/ or exp remote monitoring/ or exp telemetry/ or exp telenursing/ or exp precision medicine/ or telemonitoring.ti,ab,kw.  2 exp computer/ or exp Internet/ or exp cell phone/ or exp telephone/ or exp text messaging/ or exp electronic mail/ or exp mobile applications/ or exp smartphone/  3 (digital health or mobile health or mhealth or m-health or mobile applications or electronic health or ehealth or e-health or online or web*).ti,ab,hw,kw.  4 (wearable or wearables or mobile app or mobile apps or health app or health apps).ti,ab,kw.  5 (social networking or facebook).ti,ab,kw.  6 1 or 2 or 3 or 4 or 5  7 minority languages/ or Minority Groups/ or cultural diversity/ or Indians, North American/ or Aboriginal.mp. or first nations.mp. or Indigenous Peoples/ or Asian Continental Ancestry Group/ or "emigrants and immigrants"/ or refugees/ or "transients and migrants"/ or ethnic groups/  8 barrier*.mp. or digital divide/ or limited English proficiency/ or patient navigation.mp. or literacy.mp.  9 6 and 7 and 8  10 limit 9 to (English language and yr="2011 -Current") |
